# Wearable Devices for Environmental Monitoring in the Built Environment: A Systematic Review

**DOI:** 10.3390/s21144727

**Published:** 2021-07-10

**Authors:** Francesco Salamone, Massimiliano Masullo, Sergio Sibilio

**Affiliations:** 1 Construction Technologies Institute, National Research Council of Italy (ITC-CNR), Via Lombardia, 49, San Giuliano Milanese, 20098 Milano, Italy; 2 Department of Architecture and Industrial Design, Università degli Studi della Campania “Luigi Vanvitelli”, Via San Lorenzo, Abazia di San Lorenzo, 81031 Aversa, Italy; massimiliano.masullo@unicampania.it (M.M.); sergio.sibilio@unicampania.it (S.S.)

**Keywords:** environmental monitoring, wearable devices, wearables, systematic review, visual environmental factor, acoustic environmental factor, thermal environmental factor, air quality environmental factor

## Abstract

The so-called Internet of Things (IoT), which is rapidly increasing the number of network-connected and interconnected objects, could have a far-reaching impact in identifying the link between human health, well-being, and environmental concerns. In line with the IoT concept, many commercial wearables have been introduced in recent years, which differ from the usual devices in that they use the term “smart” alongside the terms “watches”, “glasses”, and “jewellery”. Commercially available wearables aim to enhance smartphone functionality by enabling payment for commercial items or monitoring physical activity. However, what is the trend of scientific production about the concept of wearables regarding environmental monitoring issues? What are the main areas of interest covered by scientific production? What are the main findings and limitations of the developed solution in this field? The methodology used to answer the above questions is based on a systematic review. The data were acquired following a reproducible methodology. The main result is that, among the thermal, visual, acoustic, and air quality environmental factors, the last one is the most considered when using wearables even though in combination with some others. Another relevant finding is that of the acquired studies; in only one, the authors shared their wearables as an open-source device, and it will probably be necessary to encourage researchers to consider open-source as a means to promote scalability and proliferation of new wearables customized to cover different domains.

## 1. Introduction

The so-called Internet of Things (IoT), a spider web of networked and interconnected objects that have proliferated over the past decade, could have a far-reaching impact on determining the relationship between human health and environmental quality [1] due to its ubiquitous intelligence [2].

Various studies have made it possible to thoroughly investigate the potential of IoT to improve all aspects of our lives, from industrial IoT to connected health or smart cities [3,4,5]. Among the IoT devices, the smartphone is the most widely used. In fact, the smartphone has become a disruptive presence in all essential various human activities: in some applications, the smartphone can be used to check the health of the users [6], or it can be used to acquire geographic coordinates from the real life of the users, which define the most relevant “place-of-interest”, described as “a location where the user usually goes and stays for a while” [7]. Smartphones can also be used to record information for user-profiling, and they can also influence the level of life satisfaction [8,9].

**Figure 1:**
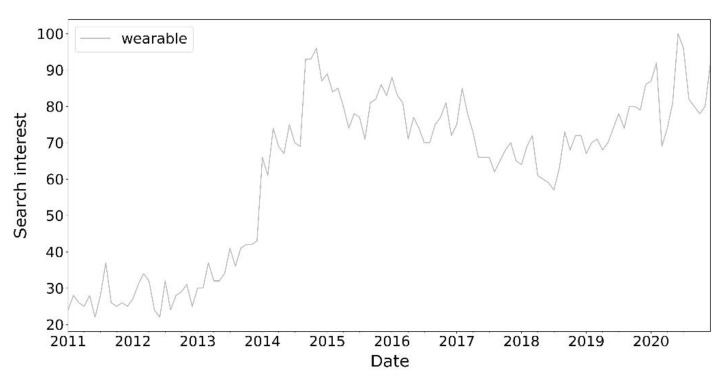
The global trend over the last 10 years of the keyword “wearable”—Google, 2020.

While smartphones have been confirmed as a ubiquitous technology that inevitably interferes with our daily lives, more and more users are also interested in using wearable devices or simply wearables. Google Trends, a service that provides accurate and representative information about users’ online search habits, reports on the global rise in consumer interest over the past decade for the keyword “wearable” (Figure 1).

Figure 1 highlights the increasing interest of consumers in wearable devices, who are probably attracted by the desire to improve the quality of life or because their behaviour may also be influenced by IT vendors who, because the smartphone market is mature, try to create new advertising campaigns [10] to create a new demand for mobile devices, focusing their attention on smartbands and smartwatches [11] without considering real needs and investing in research to prove the effectiveness of their products [12].

While there are different practical experiences with the use of wearables for ergonomics [13] or health monitoring [14,15] and workers’ safety [16], few researchers have investigated the impact of IoT-based infrastructure in environmental monitoring in the last decades. In [17], the authors systematise knowledge in the field of industrial wearables’ safety to assess the relevance of their use in enterprises as the technology maintaining occupational safety, to correlate the benefits and costs of their implementation, and, to outline promising directions for future work in this area.

In [18,19], the authors performed an extensive literature review of low-cost sensing technologies for air quality monitoring and exposure assessment and for monitoring air pollution and physical activity using sensors. In [20], the authors reviewed miniaturised sensors to measure airborne gasses and Particulate Matter (PM). In [21], the potential of different sensor-based modules for air quality monitoring was analysed in terms of power consumption, cost, response time, and lifetime. In [22], the authors presented a literature review of current research and developments on wearable devices from both academia and industry to be considered for environmental monitoring as a whole. In [23], the authors focused on low-cost IEQ sensors and cloud-based platforms to create holistic, personalized, and scalable well-being monitoring systems. They emphasised the need for wearables to create personalised approaches to IEQ monitoring.

Our study differs from the above works. It is the only one that conducts a systematic review on the four different environmental factors (visual, acoustic, thermal, and air quality) and uses the results to define the trends of research of wearables for environmental monitoring.

But, when was the term wearable introduced, and how can it be defined? What are the main considered areas of environmental monitoring? The following two sections will provide answers to these questions.

### 1.1. Introduction to Wearables

Even though the first example of clothing with smart capabilities referred to the 1980s, with an example of a shoe-based computer, designed and developed to assist gamblers in a casino [24], the term “wearable” was first used in the scientific literature in 1996 when, in [25], the author presented a personal imaging system and, in [26], where the author gave an overview of energy generation during the user’s daily activities, removing the technological limitation of batteries to power wearables. In 1997, the researchers of MIT media laboratory, Picard and Healey, used the term “affective wearables” [27] to refer to a system equipped with sensors that allowed detecting affective patterns, such as heart rate variability and electrodermal activity. To date, the class of wearable electronics or technologies, called “wearables” for short, has attracted increasing public interest and is generally identified as a category of devices that can be worn or tattooed on the human skin or even implanted in the human body to continuously and accurately monitor some variables (biometric in most of the cases, but also environmental in some other cases) without interrupting or restricting the user’s movements [28].

The United International Children’s Emergency Fund (UNICEF) also addressed the issue of wearables by proposing a competition on wearable design, with the intent of highlighting the ethical implications of wearable research. In this context, UNICEF aimed to highlight the potential of wearables to not only help consumers become more active, but also to improve their quality of life, thus promoting social interest among the public. UNICEF recommended the following requirements for potential wearables: low-cost, low power consumption, robustness and durability, scalability, and created to be open source and in the public domain [29,30].

The many commercial wearables that have entered the market in recent years do not seem to meet the above prerequisites but rather serve commercial purposes by connecting watches, glasses, jewellery, which differ from standard devices only by the term “smart” [31], to the smartphone in order to improve its functionality, enabling, for example, payment for commercial objects, monitoring of physical activity [32], and collection of personal biometric data.

### 1.2. Environmental Factors to Be Monitored

Environmental Quality (EQ) can be subdivided by analysing it in terms of Indoor EQ (IEQ) or Outdoor EQ (OEQ). Both IEQ and OEQ are important to ensure the health and well-being of people.

It is well known that users spend a large part of their time indoors, so the quality of environments within buildings and the satisfaction and well-being of occupants is a hot topic today [33].

Especially in low-cost housing, where the limited indoor space may lead occupants to spend more time doing various outdoor activities, the quality of the outdoor environment is crucial [34].

Both IEQ and OEQ refer to a holistic concept that includes various environmental factors: visual, acoustic, thermal, and air quality.

Considering the thermal aspect in indoor environments, it is possible to improve occupant satisfaction and productivity [35]. The thermal aspect is also widely considered in outdoor spaces to understand the human response to the environment, especially in the hot season when the heat island effect, defined as the phenomenon in which the temperature recorded in an urban area tends to be higher than the surrounding areas, becomes more an issue to be considered by policymakers and for which mitigation measures have to be considered [36].

The air quality factor is another important aspect to be considered, which dramatically impacts the productivity and health of users indoors [37] and outdoors [38]. The concentrations of some variables or indoor pollutants (CO_2_, VOCs, PM, NO_2_) correlate strictly with those monitored outdoors, depending on the ventilation settings and indoor or outdoor production. As can be seen, air quality is a very important issue and is often analysed through wearables.

Visual environmental factor in indoor spaces has been shown to affect mental health and productivity of occupants [39]. It could have a cross-modal or combined effect on the perception of other Environmental Effects (EFs). This is probably the only EF studied more thoroughly in indoor environments due to controlling and optimising artificial lighting systems and daylighting.

The acoustic EF has been widely studied indoors and outdoors. Many of the principles and investigations could be applied indiscriminately indoors and outdoors [40,41,42,43,44].

In all cases, monitoring all four environmental factors could be helpful to understand the complex area of interaction (cross-modal or combined) among the different environmental aspects and user perception of IEQ or OEQ. In order to link the term wearables with the environmental aspect, some new acronyms have been introduced to emphasise the class of IoT devices for monitoring some environmental factors [45]: Personal Environmental Monitoring System (PEMS), an IoT device, to measure indoor environmental exposure to contaminant [45] and Wearable Environmental Monitoring System (WEMS), usually used outdoors, which can also communicate with PEMS [45].

This article aims to answer the following main question: What is the global trend in the scientific literature regarding environmental monitoring with wearables? To this aim, a customised query was used through a specific search engine.

The following sections describe the methodology to define the query and how the acquired data were used to perform the systematic review and the most relevant scientific literature findings related to wearables and environmental monitoring. Limitations are discussed in the conclusions section, with some future improvements.

## 2. Methodology

The methodology for collecting the papers consists mainly of two steps: the identification of the database to use and the definition of the query for downloading the metadata used for the systematic review.

The first was about establishing the database from which the information was obtained. In this case, the Scopus database was selected. Among the different available databases (e.g., PubMed, Web of Science, Google Scholar), the Scopus search engine, developed by Elsevier, was used. Its resources include Institute for Scientific Information (ISI) and Scopus-indexed papers. Scopus focused primarily on the field of physical sciences, health sciences, life sciences, and social sciences [46], which is consistent with the aim of this research. The covered period is from 1966 to the present [46]. Also, this aspect is consistent with our systematic review since, as reported in the previous Section 1.1, the oldest paper presenting a “wearable” device with computational capabilities was published in the 1980s. More details about the characteristics of the main databases can be found in [46]. Scopus provides an advanced search that allows more operators and codes to make a specific query. The search results in Scopus can be exported in different formats [46]. Scopus provides all metadata as furnished by publishers for all indexed content, including authors, affiliations, the title of the paper, abstract, authors and related affiliations, year of publication, volume, issue, pages, number of citations, type of document, Digital Object Identifier (DOI).

A comprehensive and detailed search guide can be found in [47]. All defined queries can be url-encoded, saved, and a notification can be enabled to get information about newly published works matching the defined query.

Once the search domain was defined, considering the advanced search functionality of Scopus, four different queries were defined related to the specific EFs among the ones considered: thermal, visual, acoustic, and air quality. In defining the four queries, a screening process was performed before downloading the data to avoid capturing papers not in line with the review’s objective.

**Figure 2:**
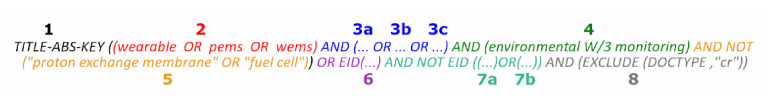
Query structure consisting of eight main sections.

The general query structure was the same for the four EFs (Figure 2).

Figure 2 shows how eight concatenated sections characterise the overall structure of the query:

1. The TITLE-ABS-KEY field code (in black) was considered so that only documents in which the selected words appear in the titles, in the abstracts, and in the keywords could be extracted and collected. In particular, the KEY field refers to a combined search that considers, among the other things, the keywords (AUTHKEY) assigned to the document by the author and the controlled vocabulary terms (INDEXTERMS) assigned to the document. Researchers sometimes pay limited attention to the definition of the AUTHKEY, even though keywords play an important role in communicating scientific results [48]. While the INDEXTERMS are added by a team of professional indexers at Scopus, based on different vocabularies [49]. In both cases, when the research area is new or changing rapidly, it is possible to highlight a highly clustered set of new keywords [50].
2. The first set of keywords (in red) was related to the overall topic of wearables, also considering the synonymous acronym PEMS (Personal Environmental Monitoring Systems) or WEMS (Wearable Environmental Monitoring Systems). The operator OR joins the three words such that at least one of the considered terms is included. This Section 2 is the same for all queries that refer to all four EFs.
3. The second set of words (in blue) was connected to the first set of keywords with the operator AND. Section three of the query is characterised by a set of targeted keywords representing each of the four EFs. In particular, Section 3a specifies physical quantities, Section 3b specifies instruments, and Section 3c specifies domains for each EF.
4. Two additional keywords, “environmental” and “monitoring” (in green), were linked to the previous sections with the operator AND. These two terms are separated by the proximity factor “w/3” indicating that they should be within three words of each other without considering the order, thus capturing the simplest form “environmental monitoring” but also more constructed forms such as “monitoring of the environmental (conditions, impacts, etc.)” or “environmental analysis and monitoring”, etc.
5. The “AND NOT” operator (in orange) was used to exclude documents that contain the term specified in the search: “proton exchange membrane” or “fuel cell”, since the acronym PEMS in Section 2 can be misleading because it can refer to a portable environmental monitoring system but also to “proton exchange membranes”, which is often associated with “fuel cell” (the related correct acronym is PEMs with lowercase “s”, but Scopus’ search engine takes into account lemmatisation, that is the process of grouping the inflected forms of a term).
6. The operator OR, associated with the acronym EID, which stands for “Electronic IDentifier” and is a unique alphanumeric string created to identify a record in Scopus, was used to add some other documents, not already considered and derived mainly from the biography screening of both other literature reviews [18,19,20,21,22,23] and the extracted papers derived from Scopus searches with query consisting of Sections 1–5 and 8 of the overall query structure here presented (Figure 2).
7. The operator AND NOT associated with EID was used to exclude some other documents that do not match the research purpose. In particular, Section 7a refers to paper excluded through the screening process performed considering the title and the abstract, while Section 7b refers to manuscripts that were not considered after full-article assessment.
8. The last part of the query (in grey) was used to exclude certain types of documents. In this sense, “cr” stands for “conference review”.

The publication year range was not specified in the overall query structure: it was directly defined by the year of availability of the papers.

Regards the inclusion (and non-inclusion) of some articles via the EID code, as performed in Sections 6 and 7, it is possible to highlight that choosing the potential eligibility or ineligibility of a study, it could be possible to affect the review by a bias [51] due to the personal decision performed by a single author. To avoid this circumstance, a labor-intensive, time-consuming, double data extraction was performed by two authors. As a result, they came to a consensus over discrepancies through discussion and in consultation with the third author, in line with the suggestion reported in [52]. In particular, the EID string provided by Scopus was used in Sections 6 and 7 to assure reproducibility and transparency of the selection criteria, in line with the requirements provided by [53].

**Figure 3:**
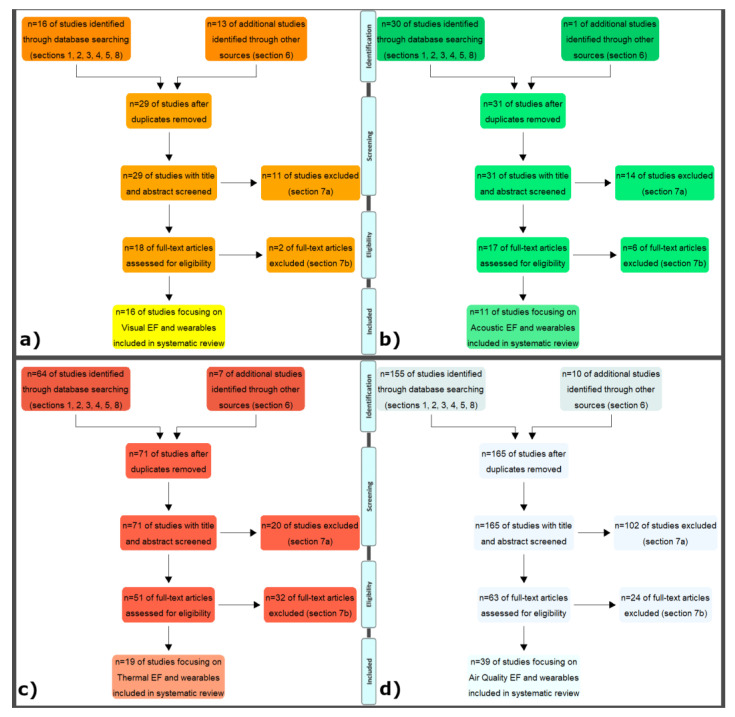
PRISMA flow diagrams: (**a**) Visual EF; (**b**) Acoustic EF; (**c**) Thermal EF; (**d**) Air Quality EF. The number of sections refers to query structure (Figure 2).

Figure 3 reports the Preferred Reporting Items for Systematic reviews and Meta-Analyses (PRISMA) [54] flow diagrams for all four EFs, allowing to present the flow of information on how studies were found, collated, and screened for systematic reviews.

It is possible to highlight how in all four EFs, the additional studies were added only if they were not acquired by Scopus search. Consequently, the duplicates papers are null and the total number of papers considered in the following steps is exactly the sum of the papers from the database search plus the additional ones derived from Section 6.

The final queries for all four EFs are reported in Appendix A.

It can be observed that Section 6 reports only one additional EID for acoustic EF, while Section 7 is quite relevant for air quality EF: this is mainly since some of the research studies report emission measurement monitored by portable systems installed on board of off-road construction equipment, trucks.

For the four queries, the number of selected documents (search performed in April 2021) is of the same order of magnitude for acoustic (11) and visual (16), as well as thermal (19) and air quality (39).

**Figure 4:**
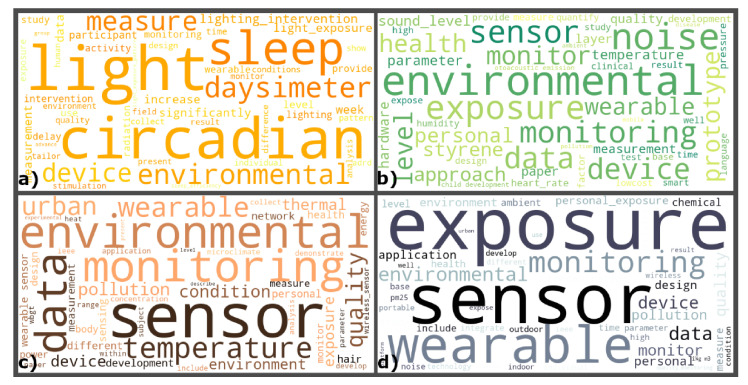
Exploratory Analysis with the word cloud of the most used terms in the title and abstract of the selected papers: (**a**) Visual EF; (**b**) Acoustic EF; (**c**) Thermal EF; (**d**) Air Quality EF.

To check the consistency of the considered terms, each of the four .csv was downloaded. Of the various available columns, only those related to the title and the abstract were considered and merged into a single column. The text in each cell of this column was pre-processed: each sentence present in each cell was tokenised, and all punctuations marks, stop words, and words less than three letters in length were removed. Then, an exploratory analysis was performed to check a visual representation of text data using a word cloud defined considering the word cloud package for Python, where the importance of each word is displayed in terms of frequency of occurrence with font size (Figure 4). For each EFs, the 50 most frequent words in the text of the title and abstract were visualised, ensuring that the screening process was performed correctly and that the selected papers were consistent with the aim of the research.

In Figure 4a, the frequent words are light, environmental, sleep, circadian, device, measure, daysimeter, the latter referring to a wearable device used to monitor the light to which the user is exposed.

In Figure 4b, among the most common words are noise, monitoring, exposure, health, wearable, environmental.

In Figure 4c, sensor, wearable, temperature, thermal, urban, and environmental are the most common words.

Finally, in Figure 4d, exposure, monitoring, wearable, sensors, and environmental are the most frequent words.

The document resulting from the search with the four queries were downloaded in “.csv” file format and considered for systematic review.

## 3. Systematic Review

The main findings resulting from the systematic review were defined separately for each of the four EFs.

### 3.1. Monitoring of Visual EF with Wearables

A total of 16 papers were considered for visual EFs, and 12 of them are based on the use of daysimeter, which was first introduced to the scientific community in 2005 [55]. In its first prototypical form, it was equipped with two sensors: a photopic sensor consisting of the Hamamatsu S1223-01 mounted in series with a subtractive glass filter and an opal glass; the other sensor was the Hammamatsu G1962 GaP, mounted in series with a colored glass filter (Schott Glass GG19) to achieve the proper short-wavelength radiation and a gel filter (Roscolux #08, Pale Gold) that provides peak sensitivity to light with a wavelength of 440 nm and is called “blue” sensor for this reason [55]. During the development of this prototype, other alternative sensors were also considered. In all other developments, the device provides the measurement of optical radiation through a glass-filtered silicon photodiode that matches the standard photopic curve and a system consisting of a short-wavelength (blue) sensor equipped with a UV-blocking glass filter that has a long-wavelength cut-off at about 580 nm and a spectral response that peaks at 460 nm. The data obtained from the device are downloaded and post-processed using the human circadian system response to light (CL_A_) model proposed in [56], which is used to define the Circadian Stimulus (CS) [57]. The board to which the photopic sensors are connected is also equipped with two orthogonally aligned accelerometers used to calculate an activity index defined as the root-mean-square deviation of acceleration in two dimensions, monitored every 30 s [57]. In a more advanced version, called Daysimeter-D, the two orthogonally oriented accelerometers have been replaced by three orthogonally oriented solid-state accelerometers to monitor the rest/activity pattern. The Daysimeter-D also allows the measurement of R, G, B, and IR channels with a peak spectral response at 615, 530, 460, and 855 nm, respectively [58]. The device was compared with other similar devices and calibrated in terms of illuminance [lx] and CLA, as reported in [59]. All described alternatives were used for indoor visual applications: laboratory, office, domestic environment, and hospital. As a practical implication, all these studies raise the need to consider a wearable system for measuring optical radiation in many applications related to human well-being, which may provide important insights into the relationship between circadian disruption [57] and well-being [60,61,62,63,64,65,66,67].

In other studies where the daysimiter is not considered, the scope of the research are different: in [68], a miniaturised microclimate station, specifically tailored to be worn while walking or cycling and therefore collecting data according to the pedestrian perspective in anthropogenic areas, was used to collect data on illuminance levels and some other variables related to thermal and air quality EFs; in [69], a calibrated wearable device, called Eco-Mini, used for environmental monitoring is presented; in [70], the authors showed how a textile composed of Janus chromic fibers shows stable practical performance and excellent sensitivity to UV/IR radiation to achieve real-time, energy-free, visual monitoring of IR radiation temperature and UV index; finally, in [71], the authors presented a fully low-cost and open-source (in accordance with the requirements defined by UNICEF, see Section 1.1) wearable light data logger for studying physiological and psychological effects of light.

Appendix B, Table A1, lists the papers that were selected and considered for this particular EF.

### 3.2. Monitoring of Acoustic EF with Wearables

A total of 11 papers were considered for acoustic EF.

In [72], the authors presented a wrist-worn device consisting of four layers: the two outermost layers are used to monitor the sound level and gasses in the environment. The two sensor layers are connected to the host layer by a layer called “flex interface” by the authors, which allows the connection of new hardware without developing a new physical layer. The main objective of this device development is to measure personal exposure to several physical (air humidity and temperature and air pressure) and chemical environmental parameters (CO and NO_2_) known to be hazardous, in addition to the sound pressure level monitored by an analogue sound sensor MLMS-EMGN-4.0. In [73,74], the same authors focused on the design and development of the case of this wearable, which was manufactured using additive manufacturing techniques. In [75], the same authors reported the development of this multi-layered wearable that can capture parameters from the environmental, behavioural, and physiological domains.

In [76], the researchers presented the NEATVIBEwear device developed for the personal measurement of exposure to ultrafine particles (UFP) and noise in a pediatric population (it was tested at home and school), allowing the independent and/or combined effect of these environmental health exposures to be studied.

In [77], the authors justify using a smartphone with the NoiseSpy application and the Empatica E4 wearable with a striking correlation between noise and heart variability in outdoor environments. Particular is the case of [78], where workers employed in molding and in artifacts finishing were equipped with a commercial wearable device (Quest DLX-1) to monitor noise exposure. Biological monitoring of styrene exposure using urine concentration was also conducted. The study revealed that the group of workers exposed to high noise level was also exposed to low styrene concentration. The group exposed to high styrene concentration near the limit of 20 ppm was also exposed to the lowest noise level. This resulted in a significant negative correlation between otoacoustic emission and styrene concentration. In [69], the calibrated portable device Eco-Mini, already mentioned in the section of Visual EF, also allowed the measurement of Sound Pressure Levels. In [79], the authors demonstrated the feasibility of collecting personal Particulate Matter (PM2.5), language, noise data, cognitive assessments, and biospecimens from a sample of 3-4-year-old children using wearable ultrafine particle sensors and LENA—Language Environment Analysis System. Two different approaches were then collected to provide an example of real-time information on urban acoustic pollution [80] and overall personal pollution [81], where the PONG device can monitor ambient sound levels as well as VOCs and NO_2_, UVA and UVB, air temperature, relative humidity and air pressure data.

Appendix B, Table A2, lists the papers that were selected and considered for this particular EF.

### 3.3. Monitoring of Thermal EF with Wearables

A total of 19 papers were considered for thermal EF.

The studies reported in [68,81] have already been presented and refer to the possibility of collecting thermal data from the pedestrian perspective in the city. In [82], the wearable enables the acquisition of representative data of urban microclimate conditions at a pedestrian level during a heatwave in a historical hilly city in central Italy. These data can be used to investigate microclimate variations within the city due to urban configuration and architectural design, human activities, and anthropogenic actions responsible for local overheating and to calculate direct thermal indices for human comfort assessment. In [83], the authors reported a research study on multi-stimuli (air temperature and UV) responsive chromism capable of displaying different colours in four different temperature ranges (blue at T < 15 °C, green at 15 °C < T < 33 °C, red at 33 °C < T < 65 °C, and white T > 65 °C) covering a wide range of applications, more than just monitoring environmental conditions. In [84], the authors used different wearables to evaluate the effects of hair exposomes in Brazil. In [85], the authors estimated Heat Exposure of public service workers in Birmingham, Alabama, using thermometers attached to the workers’ shoes. In [86], the authors present We-Safe, a self-powered sensor network system used for safety applications. In [87], the authors investigate an artificial, low-cost, skin-like temperature sensor that was highly flexible and provided for visual evaluation of temperature based on pectin and xanthan gum, which can be used in many fields such as electronic skin, human body temperature measurement, and environmental monitoring. Thus, this sensor can be used to alert people in real-time to prevent health issues resulting from extreme changes in human body temperature. In [88], the authors demonstrated the feasibility of a wearable wireless sensor system that can be attached to a uniform and used for temperature monitoring and can be activated remotely by an RF control signal. In [89], the authors presented a novel MoS_2_/Cu_2_S hybrid grown on disposable cellulose paper using a hydrothermal method to measure relative humidity and temperature, among other physical quantities. The data can be wirelessly transmitted to a smartphone with an appropriate application. In [90], the authors presented a wearable sensor for analysing personal exposure to the thermal environment and air quality (ozone, particulate matter, CO). The potential of the proposed solution is to fill the gap left by traditional air pollution monitoring. However, it still has a limitation due to the power consumption of the sensor. In [91], the authors present a smart indoor environment monitoring system for safety applications. It is based on custom wearable sensor nodes connected to a static WSN. The system is designed for a hazardous gas environment and thermal monitoring. However, it could also be used for several other safety applications or other areas, such as tracking medical devices in a hospital. In [92], a wearable system for continuous environment and health monitoring in chronic respiratory disease is presented, consisting of a wristband, a chest patch, and a handheld spirometer. In [93], the authors developed “MyPart” to address the need for a wearable device that combines accuracy, low cost, and portability in a single design. The study also reports the results of a preliminary user study conducted to evaluate the overall system’s experience with appropriate results in terms of overall performance. In [94], various subjects wore a series of instruments that recorded individual microclimatic and physiological responses along a fixed pedestrian route that passed through various urban structures. Subjects experienced different thermal environments that could not be represented by fixed-point routine observation data. A clear dependence of sweating on gender and body size was found; men sweated more than women; overweight subjects sweated more than standard/underweight subjects. The temperature of the skin (Tskin) had a linear relationship with Standard Effective Temperature and a similar clear dependence on sex and body size differences: Tskin of the higher sweating groups was lower than that of the lower sweating groups, reflecting differences in evaporative cooling by sweating. In [95], the authors report on the design and initial deployment of the Citisense mobile air quality sensing system, which collects information about the thermal environmental and ambient air quality. In [96], the authors present in-depth knowledge on sensor selection and calibration of sensors for wearable applications for thermal and air quality assessment. In [97], laboratory testing of the prototype UPAS (Ultrasonic Personal Aerosol Sampler) shows excellent agreement with equivalent samplers in the federal reference method for gravimetric analysis of PM_2.5_ over a wide range of concentrations. UPAS also monitors thermal variables (air temperature and relative humidity). In [98], thermal data of the environment and skin temperature are collected by wearable sensors and a cloud platform for monitoring environmental parameters in e-health applications. The authors suggested that this type of monitoring system is not suitable for critical situations. However, many people suffering from chronic diseases and their families can benefit from this type of system.

Appendix B, Table A3, lists the papers that were selected and considered for this particular EF.

### 3.4. Monitoring of Air Quality EF with Wearables

A total of 39 papers were selected for air quality EF.

Depending on the substance to be monitored, all these studies can be divided into four main categories. The first group consists of research studies focusing on monitoring Volatile Organic Compounds (VOCs). VOCs include an extensive family of chemical substances: from aromatic to ketones, through alcohols, aliphatic, hydrocarbons, aldehydes, ethers, and acids. They can be emitted by building materials and during cooking, and increase their concentration indoors if they are poorly ventilated [99]. On the other hand, in outdoor spaces, the primary sources of VOCs are roads traffic, fossil fuel combustion, and pesticides. Due to the vast differences among chemical substances, the effects on human health from exposure to VOCs can be diverse: from skin or eye irritation to headaches, nausea, and cancer [100]. The selected studies can be divided into those in which wearables were used only for outdoor monitoring in this category. In particular, in [101,102], the wearable “Microfabricated Preconcentrator Chip—μPC” was used to show the potential applications in occupational risk assessment for specific occupations, such as industries involving direct handling of petroleum products or in large-scale asthma population studies in pediatric and teenagers. In [68], the previously presented wearable can also measure pedestrian exposure to VOCs in cities. In [103], the authors presented WearAir, an original expressive T-shirt that detects the air quality of the wearer’s environment based on the measured volatile organic compounds. WearAir may be useful in motivating others to explore ways to communicate environmental information to lay people more effectively. The second subcategory refers to the study in which wearables were used indistinctly in indoor or outdoor environments. In [104], the authors demonstrated with a practical application the potential of the proposed wearable device to cope with many real-world analyte monitoring applications.

In [105,106], the authors presented a cost-effective and reliable platform for personal exposure assessment. Several comparisons and tests showed that the proposed VOC device is suitable to characterise personal exposure in many real-world scenarios.

In [107], the authors presented a wrist-worn Asthma Research Tool (ART) designed to identify and detect asthma triggers using only low-cost components.

In [69], a wearable called “Eco-Mini” was presented, which the authors concluded overcomes the limitations of the first generation of low-cost environmental monitors that were generally not suitable for clinical environmental health studies due to practical challenges such as calibration, reproducibility, form factor, and battery life. In [108], the proposed wearable was able to detect not only VOCs but also O_3_, NO_x_, and CO_x_.

The second group of research studies consists of those that focus on monitoring Particulate Matter (PM), also called particle pollution. PMs come in many sizes and shapes and can consist of hundreds of different chemicals. Some are emitted directly from sources, such as construction sites, dirt roads, fields, smokestacks, or fires. In contrast, others result from the atmospheric reaction of SO_2_ and NO_2_, which in turn are produced by power plants, industries, and motor vehicles [109]. This research study category can be divided into those where the corresponding wearables are mainly used indoors, and those where they are indiscriminately applied indoors or outdoors, and those that are mainly outdoors. Research studies related to the first case include [110], where a wearable called “Ultrasonic Personal Aerosol Sampler—UPAS” was used to monitor PM_2.5_ in households in Honduras where rural women use wood-fired cookstoves. In [111], the authors conducted a research study to assess the exposure of worshippers to PM_10_ and PM_2.5_ in two different types of Buddhist temples in Tai-Chung. Samples were collected using Personal Environment Monitors (PEMs) connected to personal pumps flow rates of 2 L/min. The PEMs were worn by research staff, who mimicked the activities of the worshippers to determine their exposures to PM_10_ and PM_2.5_ in both temples. To reduce exposure to PM_10_ and PM_2.5_, the authors recommend spending less time in Buddhist temples, choosing a well-ventilated temple, or avoiding visiting temples on the first and 15th days of the lunar month. In the previously presented study [76], the authors used wearables to monitor noise and assess personal exposure of UFP (ultrafine particles) in the pediatric population, allowing researchers to examine the independent and/or combined effects of these health-related environmental exposures. The same research objective is pursued in the previously presented work [79], in which, in addition to the thermal and acoustic aspect, wearables worn by 3-4-year-old children are used to collect PM_2.5_ and PM_10_ data at school and home, demonstrating that it is possible to collect personal Particulate Matter with wearables when considering this population. In the previously presented work [90], the EnviroSensor 2.0 wearable was used in addition to thermal and location data in a laboratory test to evaluate the potential of this device to collect particulate matter, ozone, and CO concentration data.

Considering the studies on indoors and outdoors PM monitoring, the authors in [112] demonstrated the feasibility of using wearables for real-time remote air quality and health monitoring applications. In [113], an experiment was conducted in Athens-Greece to collect PM_1_, PM_2.5_, and PM_10_ data. It was shown that residential air quality was determined by the type and intensity of outdoor air sources and their vertical distance from the street. Indoor activities such as cooking and cleaning further increased PM concentrations and formulated air quality, while particle accumulation was evident. In [114], personal pollution monitoring and GPS tracking were used to assess children’s PM_2.5_ exposure in their everyday environment. The overall results of this study indicate that mean PM_2.5_ exposure was lowest for children who walked to and from school and higher for those who were driven. In [115,116], the authors presented the results of a study conducted under the European Research Council funded “Cardiovascular Health effects of Air pollution in Telangana, India—CHAI” project, which investigated the association of particulate air pollution from outdoor and household sources with markers of atherosclerosis. In the previously presented study [93], the wearable “My part” was developed, distinguishing and counting particles of different sizes.

Finally, only two studies monitored PM only outdoors. In [117], a correlation was found between air quality indicators, participants’ subjective feelings about air quality, physical activity status measured with wearable sensors, and reported health symptoms. Of particular note is the result of [118]. The authors used data collected by wearables to develop a machine learning model to identify periods of cycling activity necessary for estimating the inhaled dose of chemicals.

The third group of research studies consists of those that focus on monitoring of the NO_2_ and CO. The first chemical is responsible for respiratory irritation or asthma in case of prolonged exposure [119]. While the second one, especially at elevated outdoor concentrations, may be of particular concern to people with some types of heart disease [120]. All the collected studies [45,72,75,95,121,122,123] explain the development phase, the practical test and validation of a customized wearable device for environmental monitoring.

The fourth group refers to studies focused on monitoring of CO_2_ concentration with simple wearables [124] or wearables exchanging data with a Wireless Sensor Network (WSN) [91,125] or a LoRa network [126].

Finally, among the collected research papers, five cannot be classified into the above-reported groups. In [88], the authors demonstrated a wearable wireless sensor system that can be attached to a uniform and used to monitor the combustible gas concentration and air temperature that a RF control signal can remotely activate. In [127], a wireless wearable ring-based sensor system for rapid electrochemical monitoring of explosives and nerve agent in vapor and liquid phases was presented. In [92], the wearable collects ozone concentration data among the other thermal, biometric, and activity data. It is interesting to note the case of study [84] where two wearables, the MyExposome wristband and the 2BTech Personal Ozone Monitor, were used to collect data on environmental aggressors (Polycyclic aromatic hydrocarbons—PAHs, oxygenated PAHs—OPAHs, Polychlorinated biphenyls—PCBs, Pesticides, organophophorous flame retardants—OPFRs, Surface ozone content—SOC) associated with hair damage. In [78], a significant negative correlation was found between the otoacoustic emission levels and the concentration of the styrene urinary metabolites, in workers employed in molding and in artifacts refining.

Appendix B, Table A4, lists the papers that were selected and considered for this particular EF.

## 4. Discussion and Conclusions

**Figure 5:**
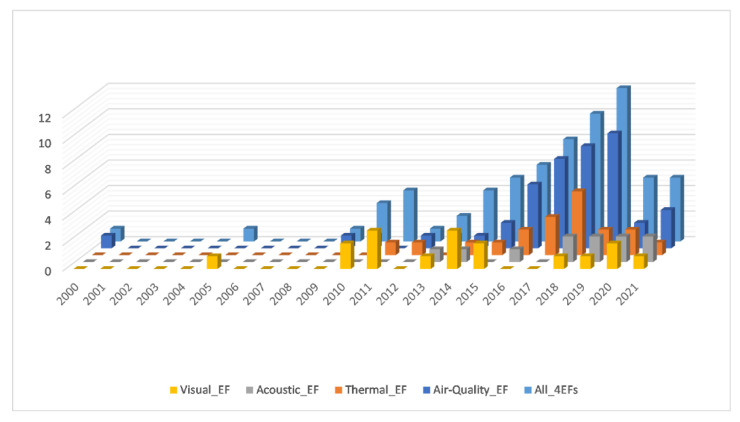
Annual Scientific production—Scopus, 2021.

Considering the Scopus functionality for combining search queries, a total of 68 papers can be summarised by entering the list number of each stored search query and the operator OR, some of which cover more than one EF. Figure 5 shows the annual scientific production for each EF and all 4EFs. In this last case, the papers that consider wearables used for multiple EFs are counted only once. The period of publication is between 2000 and 2021. The first paper published in 2000 is related to the air quality research study conducted to assess the exposure of worshippers to PM_10_ and PM_2.5_ in two different types of Buddhist temples in Tai-Chung [111]. The second one, published in 2005, refers to visual EF when the daysimeter was first introduced to the scientific community [55].

Even though the systematic review covers the last 20 years, it can be highlighted from Figure 5 how only from 2012 onwards the most significant increase in production was recorded, with an annual growth rate of 12.18% considering all 4EFs.

Of the 68 papers, most consider wearables that can monitor various EFs. The wearables used to assess visual aspects focused mainly on indoor environments. Several studies looked at the impact of daylight on circadian rhythms.

As mentioned above, wearables used for visual EF were mainly focused on using daysimeters in different contexts or, alternatively, in defining the UV, IR, or illuminance values. Probably, in the future, studying the feasibility of using some type of wearable spectrometer for visual EF assessment could be considered, allowing the effects of visual aspects in the built environment to be examined from a human-centered perspective, more than has already been done.

Acoustic EF studies have mainly focused on investigating the health effects of combined acoustic and environmental air exposures. In a case study [77], a smartphone was used as a wearable monitoring device to understand the influence of noise on heart rate variability. The main problem in this perspective is accuracy across devices: different devices will have different microphones with different sensitivity levels, making measurements vary from device to device [128]. However, a more combined integration between physical wearables equipped with different sensors and a smartphone-based application also developed using an open-source framework [129], and integrated hardware could be interesting.

While for thermal EF, the use of wearables to assess outdoor comfort could be considered widespread, for example, tying the use of these wearables to specific outdoor conditions such as the Urban Heat Island and associated impacts on human well-being; instead, it is usually used in combination with some others to determine the overall environmental conditions while conducting the test.

Undoubtedly, among the four considered EFs, air quality is the most considered in the use of wearables, and the case studies span multiple domains, as seen in Section 3.4.

As the presented review shows, the data collected by wearables can be used to define the spatio-temporal propagation of environmental parameters. In some cases, these data are combined with subjective biometric data to identify a potential pattern. In a small number of cases, the environmental and biometric data are combined with subjective feedback. This aspect could probably be explored more deeply in the near future, in a human-centric approach. As mentioned in introduction, wearables have also been used to acquire data and assess physical ergonomic risk factors, particularly on unfavourable postures as reported in [13] where it was focused on the design principles of wearables from an ergonomic perspective as one of the main future directions that this type of device could address.

In healthcare monitoring [14], wearables have been used in research studies for monitoring chronic diseases such as asthma, cardiovascular diseases, diabetes and nutrition, gait and fall, neurological diseases, stress. In [15], it was pointed out that wearable devices used for health monitoring will face several limitations due to practical difficulties in achieving user-friendly solutions, security and privacy issues, and lack of industry standards.

In [16], it was emphasised that, although a considerable research effort has been devoted to the benefits of wearables in the work environment, less attention has been paid to the empirical analysis of employees’ acceptance of wearable technology.

Ergonomic design principles, user-friendly solutions, and user acceptance of wearable technology are three main aspects that can be highlighted as the major directions that wearables used for environmental monitoring will also need to consider, more than it already has been done. From the table in Appendix B, there are no clear indications of the limitations of the proposed devices. However, after reading and viewing the photos reported in the papers, these wearables could be likely to be better integrated with clothing and apparel.

In 60% of the total cases, the accuracy of the tested system was performed simply by correlating monitored data with those acquired with calibrated sensors of a reference instrument. The percentages are quite different if each EF is considered separately: In 87% of the cases, a comparison or a calibration was performed for wearables used for monitoring visual aspects, while the accuracy of wearables used in the acoustic and thermal domain is verified in 45% of the cases. Even lower is the case of air quality monitoring using wearables, with only 40% of the tested devices. This is a limitation of the proposed wearables because a test of accuracy, if done consistently and in all cases, may reduce much of the variability and limitations of not-calibrated hardware. For all the four EFs, the devices are generally characterized by great usability when using the hardware while performing the monitoring campaign. The usability of software used for data extraction and analysis is not contemplated because it is performed in all the cases by specified researchers and not by direct involvement of participants who generally are not even aware of the measured value of the general environmental variable.

There is one point that did not emerge in this review paper that we would like to highlight as food for thought for likely future studies: related to one of UNICEF’s requirements for this type of device (Section 1.1): of the 68 studies, how many fully shared their wearable to encourage dissemination by releasing it in open-source form? The answer is as disappointing as ever: only one. Work should be done in this direction, encouraging the proliferation of open-source wearables, to promote greater scalability and the proliferation of several devices applicable in different domains. In this sense, countries that are distinguished for their technologically advanced economy and society are reconsidering their education policy and are transferring funding to promote STEM education, an acronym that integrates the academic disciplines of Science, Technology, Engineering, and Mathematics [130], and probably, considering the investigated topic of wearables for environmental monitoring, especially when released under an open-source license, it could encourage both the development of more environmentally conscious society on the one hand and the spread of coding and programming in schools on the other [131].

Following the same principles of dissemination of information in an open and shared way, we decided to publish in an open-source journal, despite not having received funding for the research carried out. We have also shared the queries used in this systematic review (see Appendix A) to verify, in the future, how the studies in this specific area of research will evolve.

This is a future and distinct research direction for improving a widespread wearable device. We hope that this systematic review will highlight the current state of innovative work in wearable natural checking and provide rules to improve further the design and development of this type of device and related practical applications.

## Data Availability

Not applicable.

## Appendix A

- Visual (16 resulting papers from 2005 to 2021): TITLE-ABS-KEY ((wearable OR pems OR wems) AND (daylight OR “color temperature” OR lighting OR luminance OR glare OR “dosimeter” OR “lux meter” OR “light meter” OR “color meter” OR colorimeter OR “luminance meter” OR photometry OR visual) AND (environmental W/3 monitoring) AND NOT (“proton exchange membrane” OR “fuel cell”)) OR EID (2-s2.0-27344452309 OR 2-s2.0-77956754851 OR 2-s2.0-85042561689 OR 2-s2.0-79957588325 OR 2-s2.0-84879967631 OR 2-s2.0-77953632392 OR 2-s2.0-81255124342 OR 2-s2.0-79952959932 OR 2-s2.0-84928173018 OR 2-s2.0-84960113897 OR 2-s2.0-84918811426 OR 2-s2.0-84907667703 OR 2-s2.0-85096669247) AND NOT EID ((2-s2.0-41849089305 OR 2-s2.0-85047120790 OR 2-s2.0-85052730774 OR 2-s2.0-84933505542 OR 2-s2.0-85044290759 OR 2-s2.0-84885042666 OR 2-s2.0-84952011313 OR 2-s2.0-85066239722 OR 2-s2.0-85087571429 OR 2-s2.0-85067399950 OR 2-s2.0-84855470006) OR (2-s2.0-85030837996 OR 2-s2.0-85086035889)) AND (EXCLUDE (DOCTYPE, “cr”))
- Acoustic (11 resulting papers from 2013 to 2021): TITLE-ABS-KEY ((wearable OR pems OR wems) AND (“sound pressure” OR “noise” OR soundscape OR loudness OR “sound level meter” OR sound OR acoustic) AND (environmental W/3 monitoring) AND NOT (“proton exchange membrane” OR “fuel cell”)) OR EID (2-s2.0-85101714199) AND NOT EID ((2-s2.0-84055184752 OR 2-s2.0-85074627122 OR 2-s2.0-85038856331 OR 2-s2.0-85102682462 OR 2-s2.0-37549066979 OR 2-s2.0-84885042666 OR 2-s2.0-84929223038 OR 2-s2.0-85045751469 OR 2-s2.0-84991109042 OR 2-s2.0-85069766055 OR 2-s2.0-85087571429 OR 2-s2.0-85068142712 OR 2-s2.0-85066081213 OR 2-s2.0-85097010457) OR (2-s2.0-85010934316 OR 2-s2.0-84984935795 OR 2-s2.0-84955466350 OR 2-s2.0-85015726258 OR 2-s2.0-85052392384 OR 2-s2.0-85086035889)) AND (EXCLUDE (DOCTYPE, “cr”))
- Thermal (19 resulting papers from 2011 to 2021): TITLE-ABS-KEY ((wearable OR pems OR wems) AND (“air temperature” OR “relative humidity” OR “radiant temperature” OR “air velocity” OR “wind velocity” OR “solar radiation” OR thermometer OR “temperature sensor” OR “humidity sensor” OR “Thermo Hygrometer” OR “Globe-Thermometer” OR “black globe temperature sensor” OR “anemometer” OR pyranometer OR thermal) AND (environmental W/3 monitoring) AND NOT (“proton exchange membrane” OR “fuel cell”)) OR EID (2-s2.0-79960211999 OR 2-s2.0-84991057252 OR 2-s2.0-84990882596 OR 2-s2.0-84864975130 OR 2-s2.0-85012992224 OR 2-s2.0-85025664819 OR 2-s2.0-85041373985) AND NOT EID ((2-s2.0-85044576922 OR 2-s2.0-85102274461 OR 2-s2.0-85087280469 OR 2-s2.0-85042584638 OR 2-s2.0-79959682725 OR 2-s2.0-85101604122 OR 2-s2.0-84962616057 OR 2-s2.0-85067457161 OR 2-s2.0-85009960940 OR 2-s2.0-84891667975 OR 2-s2.0-85050642807 OR 2-s2.0-85021806905 OR 2-s2.0-85054856073 OR 2-s2.0-85100893557 OR 2-s2.0-0032856815 OR 2-s2.0-85099112380 OR 2-s2.0-84984696179 OR 2-s2.0-85089441482 OR 2-s2.0-85079714972 OR 2-s2.0-84959878599 OR 2-s2.0-85101108646 OR 2-s2.0-85103300483 OR 2-s2.0-85103941705 OR 2-s2.0-85096397030 OR 2-s2.0-85084811022 OR 2-s2.0-85086035889) OR (2-s2.0-85090275198 OR 2-s2.0-85079494843 OR 2-s2.0-85090949175 OR 2-s2.0-85058283726 OR 2-s2.0-85053684980 OR 2-s2.0-85053504654 OR 2-s2.0-85055675218 OR 2-s2.0-85034043945 OR 2-s2.0-85029159022 OR 2-s2.0-85027702156 OR 2-s2.0-85014596472 OR 2-s2.0-85037707375 OR 2-s2.0-84994102143 OR 2-s2.0-84974605210 OR 2-s2.0-85100591108 OR 2-s2.0-84934900174 OR 2-s2.0-84943615704 OR 2-s2.0-84896698814 OR 2-s2.0-70349666850 OR 2-s2.0-58149381900 OR 2-s2.0-41849089305 OR 2-s2.0-34347376912 OR 2-s2.0-85077977296 OR 2-s2.0-85076783804 OR 2-s2.0-85072645464 OR 2-s2.0-84991661572)) AND (EXCLUDE (DOCTYPE, “cr”))
- Air Quality (39 resulting papers from 2000 to 2021): TITLE-ABS-KEY ((wearable OR pems OR wems) AND (“CO2” OR “carbon dioxide” OR “VOCs” OR “volatile organic compounds” OR “NOx” OR “nitrogen oxides” OR “SOx” OR “sulphure oxides” OR “PM” OR “particulate matter” OR “concentration sensor” OR “air quality” OR pollution) AND (environmental W/3 monitoring) AND NOT (“proton exchange membrane” OR “fuel cell”)) OR EID (2-s2.0-84991057252 OR 2-s2.0-84990882596 OR 2-s2.0-84864975130 OR 2-s2.0-77950842815 OR 2-s2.0-85012992224 OR 2-s2.0-85019211807 OR 2-s2.0-85025664819 OR 2-s2.0-85032633215 OR 2-s2.0-85041373985 OR 2-s2.0-84978821913) AND NOT EID ((2-s2.0-84891667975 OR 2-s2.0-84991628256 OR 2-s2.0-79951809148 OR 2-s2.0-34548598391 OR 2-s2.0-85084482031 OR 2-s2.0-84865570477 OR 2-s2.0-84934900174 OR 2-s2.0-85103821669 OR 2-s2.0-79952613338 OR 2-s2.0-85059670087 OR 2-s2.0-85092552189 OR 2-s2.0-85045622832 OR 2-s2.0-84860692258 OR 2-s2.0-84930205795 OR 2-s2.0-72849107023 OR 2-s2.0-84942599257 OR 2-s2.0-0042123766 OR 2-s2.0-85083203437 OR 2-s2.0-85021741462 OR 2-s2.0-84880273197 OR 2-s2.0-84868228523 OR 2-s2.0-85017422700 OR 2-s2.0-85045234275 OR 2-s2.0-79960504035 OR 2-s2.0-84991661572 OR 2-s2.0-37549066979 OR 2-s2.0-0033626567 OR 2-s2.0-50849117829 OR 2-s2.0-85064530053 OR 2-s2.0-84958243072 OR 2-s2.0-85084696209 OR 2-s2.0-85097756884 OR 2-s2.0-85028048241 OR 2-s2.0-85053816691 OR 2-s2.0-85034962458 OR 2-s2.0-0035186649 OR 2-s2.0-85042029752 OR 2-s2.0-85060148578 OR 2-s2.0-85075976987 OR 2-s2.0-0032856815 OR 2-s2.0-85021647526 OR 2-s2.0-85100893557 OR 2-s2.0-85073126584 OR 2-s2.0-85092086462 OR 2-s2.0-80054756044 OR 2-s2.0-85085695131 OR 2-s2.0-84984935795 OR 2-s2.0-85044659217 OR 2-s2.0-85009268800 OR 2-s2.0-26244441016 OR 2-s2.0-84955466350 OR 2-s2.0-84978821913 OR 2-s2.0-85091572416 OR 2-s2.0-85066239722 OR 2-s2.0-85094163061 OR 2-s2.0-84890910781 OR 2-s2.0-0029825012 OR 2-s2.0-85015715897 OR 2-s2.0-34347229727 OR 2-s2.0-85098538986 OR 2-s2.0-85010934316 OR 2-s2.0-0034164368 OR 2-s2.0-85084811022 OR 2-s2.0-85084471284 OR 2-s2.0-84994174400 OR 2-s2.0-77956922071 OR 2-s2.0-0033179964 OR 2-s2.0-20444468078 OR 2-s2.0-70449564549 OR 2-s2.0-84900342694 OR 2-s2.0-24344499484 OR 2-s2.0-84953404270 OR 2-s2.0-84898682763 OR 2-s2.0-85077723989 OR 2-s2.0-85098056476 OR 2-s2.0-84870825370 OR 2-s2.0-0033207046 OR 2-s2.0-85090275198 OR 2-s2.0-85086135361 OR 2-s2.0-84885225815 OR 2-s2.0-85049913644 OR 2-s2.0-85064598441 OR 2-s2.0-85050642807 OR 2-s2.0-85054856073 OR 2-s2.0-85041278840 OR 2-s2.0-84991109042 OR 2-s2.0-84964470801 OR 2-s2.0-85061341557 OR 2-s2.0-79951790941 OR 2-s2.0-85101690645 OR 2-s2.0-0032261477 OR 2-s2.0-85015726258 OR 2-s2.0-85039737118 OR 2-s2.0-43049149412 OR 2-s2.0-84922212195 OR 2-s2.0-77954398091 OR 2-s2.0-84994102143 OR 2-s2.0-84864283881 OR 2-s2.0-84947241573 OR 2-s2.0-85089441482 OR 2-s2.0-85064164959 OR 2-s2.0-85053439823) OR (2-s2.0-85076971711 OR 2-s2.0-85075795237 OR 2-s2.0-85071043860 OR 2-s2.0-85086035889 OR 2-s2.0-85074049696 OR 2-s2.0-78650619960 OR 2-s2.0-0034849917 OR 2-s2.0-0036306120 OR 2-s2.0-0025717462 OR 2-s2.0-85077977296 OR 2-s2.0-85077051133 OR 2-s2.0-34250637151 OR 2-s2.0-85042542326 OR 2-s2.0-85013042267 OR 2-s2.0-84994468982 OR 2-s2.0-84883570280 OR 2-s2.0-85076783804 OR 2-s2.0-85089002492 OR 2-s2.0-85052392384 OR 2-s2.0-85096397030 OR 2-s2.0-85058283726 OR 2-s2.0-85046162925 OR 2-s2.0-85104610177 OR 2-s2.0-85052751178)) AND (EXCLUDE (DOCTYPE, “cr”))

## Appendix B

**Table A1:** Selected papers for visual EF.

Reference Number of the Research Study	Visual Physical Variables Monitored (in Brackets the Name of the Used Wearable, if Available)	Other Factors Monitored (in Brackets the Name of Used Wearable if Available)	Indoor/Outdoor and Field of Application	Practical Implication	Limitation of the Considered Wearable
[68]	Lighting [lx]	Thermal, Air quality	Outdoor. Wearable used to study pedestrians’ exposure to urban environmental conditions	A miniaturized microclimate station, specifically tailored to be worn while walking or biking	
[60]	Circadian stimulus (Daysimeter)		Indoor. Office sites	CS 0.3 during daytime hours is associated with an acute alerting effect on office workers	
[61]	Personal light exposures: illuminance, circadian light (CLA), and circadian stimulus (CS) levels (Daysimeter)	Interdaily Stability (IS) and Intradaily Variability (IV)	Indoor. Household environment	The lighting intervention significantly increased circadian entrainment and significantly reduced symptoms of depression in the participants with ADRD	
[62]	Personal light exposures: illuminance, circadian light (CLA), and circadian stimulus (CS) levels (Daysimeter)	Biometric monitoring (Actigraph)	Indoor. Household environment	Flashing blue light delivered through closed eyelids during sleep can change the circadian phase, thus allowing to promote sleep health in those suffering from circadian sleep disorders.	
[58]	Personal light exposures: illuminance, circadian light (CLA), and circadian stimulus (CS) levels (Daysimeter)	Rest/activity monitoring with three orthogonally oriented, solid-state accelerometers (Daysimeter)	Indoor. Household environment	Circadian phase changes resulting from a light intervention are consistent with those predicted by previously published Phase Response Curves [132]	
[63]	Personal light exposures: illuminance, circadian light (CLA), and circadian stimulus (CS) levels (Daysimeter)		Indoor.	A lighting intervention, tailored to increase daytime circadian stimulation, can be used to increase sleep quality and improve behavior in patients with ADRD	
[59]	Personal light exposures: illuminance, circadian light (CLA), and circadian stimulus (CS) levels (Daysimeter)		Indoor/Outdoor		
[64]	Photopic illuminance in lux from 400 to 900 nm (Philips Actiwatch-L) and photopic illuminance in lux from 400 to 700 nm (Daysimeter)		Indoor. Hospital	Study describes the difference between light levels measured at the wrist (Actiwatch-L)	
[65]	Personal circadian and photopic light exposures (Daysimeter)	Acceleration and temperature data (Daysimeter)	Indoor. School	Not significant difference between two groups, one of which was wearing orange glasses	
[66]	Photopic illuminance in lux from 400 to 900 nm (Philips Actiwatch-L) and photopic illuminance in lux from 400 to 700 nm (Daysimeter)		Indoor. Hospital (day-shift nurse)		
[57]	Photopic illuminance in lux from 400 to 900 nm (Philips Actiwatch-L) and photopic illuminance in lux from 400 to 700 nm (Daysimeter)		Indoor. Hospital	These measurement and analysis techniques may provide important insights into the relationship between circadian disruption and well-being.	
[67]	Personal light exposures (illuminance, circadian light (CLA), and circadian stimulus (CS) levels) (Daysimeter)	Biometric monitoring (Actigraph)	Indoor. Home	This case study provides beginning support for the use of the daysimeter in the home	
[55]	Personal light exposures (illuminance, circadian light (CLA), and circadian stimulus (CS) levels) (Daysimeter)		Indoor. Laboratory and Home	A system of measurement of optical radiation for the circadian system is essential in many applications concerning human wellbeing	
[69]	Ambient light color balance (Eco-Mini)	Ozone, Sulfur Dioxide, Volatile Organic Compounds, humidity, temperature, and sound level, 3-axis accelerometer and GPS location (Eco-Mini)	Indoor/Outdoor	The presented wearable environmental monitoring, called “Eco-Mini”, overcomes pratical challenges (calibration, reproducibility, form factor, and battery life) and can be used in clinical studies.	
[70]	UV light and IR radiation		Indoor/Outdoor	The fibers and their textiles showed stable practical performance and outstanding sensitivity to UV/IR radiation to achieve energy-free, real-time visual monitoring of the IR radiation temperature, and UV index.	
[71]	UV Light, light intensity [lx], color temperature [K], and color components		Indoor/Outdoor	Practical value within disciplines such as environmental and health psychology, which seek to relate psychological outcomes to environmental exposure. Completely open-source allowing replicability.	

**Table A2:** Selected papers for acoustic EF.

Reference Number of the Research Study	Acoustic Physical Variables Monitored (in Brackets the Name of Used Wearable, if Available)	Other Factors Monitored (in Brackets the Name of Used Wearable, if Available)	Indoor/Outdoor and Field of Application	Practical Implication	Limitation of the Considered Wearable
[72]	Sound pressure level [db] (MLMS-EMGN-4.0)	Thermal: air humidity, temperature. Air quality: CO and NO_2_. Air pressure	Indoor. Laboratory	This device measures several physical and chemical environmental parameters, which are known to be hazardous.	
[76]	Noise exposure (NEATVIBEwear)	Air quality: ultrafine particles (UFP, <100 nm diameter)	Indoor. Transit, home, school	Personal measurements of UFP (ultrafine particle) and noise will enable researchers to investigate the independent and/or joint-effects of these health-relevant environmental exposures.	NEATVIBEwear, in its current release, does not allow for 1/3-octave band or spectral measurements of traffic noise.
[73]	Sound pressure level [db]	Thermal: air humidity, temperature. Air quality: CO and NO_2_. Air pressure. Motion activity (9 Degree of Freedom(DoF)) motion tracking by accelerometer (KX122-1037), gyroscope (KXG03-1034) and magnetometer (KMX62-1031).	Indoor/Outdoor	This device measures several physical and chemical environmental parameters, which are known to be hazardous. The wearable solution may overcome the issue of stationary climate sites, which are rare in cities due to their size, cost, and maintenance.	
[74]	Sound pressure level [db]	Thermal: air humidity, temperature. Air quality: CO and NO_2_. Air pressure. Motion activity (9 Degree of Freedom(DoF)) motion tracking by accelerometer r (KX122-1037), gyroscope (KXG03-1034) and magnetometer (KMX62-1031).	Indoor/Outdoor	This device is measuring several physical and chemical environmental parameters, which are known to be hazardous. The wearable solution may overcome the issue of stationary climate sites, which are rare in cities due to their size, cost, and maintenance.	
[77]	Smartphone with NoiseSpy application	Heart Rate Variability (Empatica E4)	Outdoor	The study shows the preliminary results of user study with early data analysis results, suggesting a noticeable relationship between noise and heart variability.	
[78]	Noise levels (Quest DLX-1)	Biological monitoring in saliva and in urine to evaluate the exposure to styrene	Indoor. Workers employed in molding and in artifacts refining	A significant negative correlation was found between the otoacoustic emission levels and the concentration of the styrene urinary metabolites. Otoacoustic emissions, and particularly distortion products, were able to discriminate the exposed workers from the controls, providing also a rough estimate of the slope of the dose-response relation between otoacoustic levels and styrene exposure	
[69]	Sound pressure level (Eco-Mini)	Ozone, Sulfur Dioxide, Volatile Organic Compounds, humidity, temperature, and ambient light color balance, 3-axis accelerometer and GPS location (Eco-Mini)	Indoor/Outdoor	The presented wearable environmental monitoring, called “Eco-Mini”, overcomes practical challenges (calibration, reproducibility, form factor, and battery life) and can be used in clinical studies.	
[79]	Noise [db] (LENA-Language Environment Analysis System)	Thermal: air humidity, temperature. Air quality: PM_2.5_ an PM_10_. (PLANTOWER PM5003).	Indoor. School, Home.	Preliminary results indicate that it is feasible to gather personal Particulate Matter (PM2.5), language, and noise data, cognitive assessments, and biospecimens from a sample of 3-4-year-old children.	
[75]	Sound level [db]	Air quality: NO_2_, CO. Thermal: air temperature and relative humidity. UV. Biometrics: skin temperature, heart rate. Motion activity (9 Degree of Freedom(DoF)) motion tracking by accelerometer, gyroscope and magnetometer.	Indoor/Outdoor	Wrist-worn devices can integrate parameters from the environmental, behavioral, and physiological domain	
[80]	Noise level [db]		Outdoor	The main function of the proposed study is to provide an example of real-time information on the acoustic impact on the city.	This work is currently in progress at this stage of the project so that calibration and adjustment of the final acoustic sensor can be carried out
[81]	Ambient sound level (PONG)	Air quality: VOC and NO_2_. Visual: UVA and UVB. Thermal: air temperature, relative humidity. Air pressure. (PONG)	Outdoor	The developed device has the potential to serve as a personal pollution monitor.	Further work is required to investigate the accuracy of the data

**Table A3:** Selected papers for thermal EF.

Reference Number of The Research Study	Thermal Physical Variables Monitored (in Brackets the Name of Used Sensor or Wearable, if Available)	Other Factors Monitored (in Brackets the Name of Used Sensor or Wearable, if Available)	Indoor/Outdoor and Field of Application	Practical Implication	Limitation of the Considered Wearable
[81]	Air temperature [°C], Relative Humidity [%]. (PONG).	Air Quality: VOCS and CO_2_. Visual: UV. Acoustic: ambient sound levels. Air pressure. (PONG).	Outdoor	The developed device has the potential to serve as a personal pollution monitor.	
[82]	Air Temperature [°C], Relative Humidity [%], Wind Direction [°], Wind Speed [m/s]	Air quality: VOCs [kOhm], CO_2_ [ppm]. Visual: Solar radiation [W/m2]. Air pressure [hPa].	Outdoor.	The data are representative of intra-urban microclimate conditions at a pedestrian height during a heat wave in a historical hilly town located in central Italy. These data can be used to investigate microclimate variation within the city imputable to urban configuration and architectural layout, human activity, and anthropogenic actions responsible for local overheating, and to compute direct thermal indexes for human comfort evaluation.	
[83]	Temperature [°C]	Visual: UV	Outdoor.	This unit can demonstrate different colors in four different temperature ranges (blue at T < 15 °C, green at 15 °C < T < 33 °C, red at 33 °C < T < 65 °C, and white T > 65 °C	Due to the limitation of the reaction chemistry, it’s usually very difficult to change these color change styles for better stimuli sensing performance
[84]	Air Temperature and relative humidity (Model HTR-170)	Visual: UV (UV dosimeter badge). Air quality: Polycyclic aromatic hydrocarbons—PAHs, oxygenated PAHs—OPAHs, Polychlorinated biphenyls—PCBs, Pesticides, organophophorous flame retardants—OPFRs. (MyExposome); surface ozone content—SOC (2BTech Personal Ozone Monitor)	Outdoor.	Polycyclic aromatic hydrocarbons (PAH) indicate possible causes of hair damages. This is the first meteorotropic study of its kind, combining environmental aggressors related to hair damage, opening new research hypothesis and further studies on exposome.	
[85]	Thermometers clipped on workers’ shoes (HOBO Pendant or iButton DS1922L)		Indoor/Outdoor.	Temperature from wearable thermometers, together with meteorological data, can serve as an additional method to identify occupational heat stress exposure and recommend work–rest schedules.	
[86]	Air Temperature and relative humidity (BME680 mounted on a WE-Safe wearable sensor node)	UV (si1145 mounted on a WE-Safe wearable sensor node)	Indoor/Outdoor.	The proposed sensor network system presents a good example of an Internet of Things (IoT) platform in health care and safety applications.	
[87]	Temperature [°C]		Indoor/Outdoor.	This study investigates an artificial low-cost skin-like temperature sensor that was highly flexible and allowed for visual evaluation of the temperature. It can be used to provide real-time warnings to people for preventing any health issues resulting from extreme changes in human body temperature.	
[68]	Air Temperature [°C], Relative Humidity [%] (BME280). Wind Direction [°], Wind Speed [m/s] (LCJ-CAPTEURS CV07-OEM). Surface Temperature [°C] (Flir vue Pro R)	Air quality: CO (DDScientific GD) and CO_2_ (Dynament MSH-P-CO_2_/NC) concentration [ppm], VOCs [kOhm] (Figaro TGS8100). Visual: Illuminance [lx], Global solar radiance [W/m^2^] (SP-215)	Outdoor. Wearable used to study pedestrians’ exposure to urban environmental conditions	A miniaturized microclimate station, specifically tailored to be worn while walking or biking and therefore to collect data according to the pedestrian perspective in anthropized areas.	
[88]	Air Temperature [°C] (ATxmega16E5 in-built temperature sensor)	Combustible gas concentration (NTC IGD)	Indoor. Employee state monitoring	In this work, the authors demonstrated a wearable wireless sensor system, which can be attached to a uniform and used for monitoring combustible gas concentration and temperature. The main feature of the wireless system is the possibility to be activated remotely by an RF control signal.	
[89]	Temperature [°C], Relative Humidity [%].	Breath sensing and ethanol adulteration	Indoor/Outdoor.	A novel MoS2/Cu2S hybrid grown on disposable cellulose paper by the hydrothermal method is reported for its utilization in humidity, temperature and breath sensing, and ethanol adulteration wherein the data can be wirelessly transmitted to a smartphone with dedicated application.	
[90]	Temperature [°C], Relative Humidity [%] (EnviroSensor 2.0).	Air quality: ozone, particulate matter, CO. Position: Latitude and longitude (EnviroSensor 2.0).	Laboratory test	Portable air quality sensors have the potential to fill in the gap left by traditional air pollution monitoring.	Power draw of the sensor
[97]	Air temperature [°C], Relative Humidity [%]. (BME280 mounted on the ultrasonic personal aerosol sampler—UPAS)	Air pressure [hPa] (BME280). Visual: UV, IR (SI1145-A10-GMR). Mass air flow (Omron D6F). Air Quality: PM2.5 Accelerometer/Magnetometer (LSM303DLHCTR) (UPAS).	Laboratory test	Laboratory tests of the UPAS prototype demonstrate excellent agreement with equivalent federal reference method samplers for gravimetric analysis of PM2.5 across a broad range of concentrations.	
[91]	Temperature [°C], Relative Humidity [%] (SHT11)	Air quality: CO_2_ [ppm]	Indoor.	The system based on custom wearable sensor nodes, connected to a static WSN has been developed for a hazardous gas environment, but could be applied to a number of other safety applications or in other areas, such as the tracking of medical devices in a hospital.	
[92]	Temperature [°C], Relative Humidity [%].	Air quality: Ozone. Biometric parameters: Herat rate, respiratory rate and expiratory airflow, skin impedance. Acceleration.	Indoor.	The system consists of a wristband, a chest patch, and a handheld spirometer.	
[93]	Temperature [°C], Relative Humidity [%] (MyPart).	Air quality: distinguishing and counting differently sized particles (MyPart)	Indoor/Outdoor.	The study reports the results of a preliminary user study conducted to evaluate the experience of using a new PEMS for air monitoring.	
[98]	Temperature [°C], Relative Humidity [%] (DHT11).	Body temperature [°C] (TMP102).	Indoor. People suffering from chronic diseases.	This type of monitoring systems are not suitable for critical situations, but many people suffering from chronic diseases and their families can benefit from this type of system.	
[94]	ambient temperature, humidity, wind speed (U) and short/long-wave radiation (S and L)	Physio-psychological parameters: skin temperature, pulse rate, subjective thermal sensation and state of body motion.	Outdoor.	A clear dependence of sweating on gender and body size was found; males were found to sweat more than females; overweight subjects sweat more than standard/underweight subjects. Tskin had a linear relationship with SET and depended on gender and body size differences. Tskin of the higher-sweating groups was lower than that of the lower-sweating groups, reflecting differences in evaporative cooling by perspiration.	
[95]	Temperature [°C], Relative Humidity [%].	Air quality: N02, 03 and CO	Outdoor.	Author reports the design and initial deployment of the Citisense mobile air quality sensing system.	
[96]	Temperature [°C]	Air Quality: O_2_, CO and CO_2_	Laboratory test	This paper presents in-depth knowledge on sensor selection and calibration.	

**Table A4:** Selected papers for air quality EF.

Reference Number of the Research Study	Air Quality Physical Variables Monitored (in Brackets the Name of Used Sensor or Wearable, if Available)	Other Factors Monitored (in Brackets the Name of Used Sensor or Wearable, if Available)	Indoor/Outdoor and Field of Application	Practical Implication	Limitation of the Considered Wearable
[101]	14 volatile organic compounds (Microfabricated Preconcentrator Chip—μPC)	Thermal: temperature and humidity data. GPS. (Microfabricated Preconcentrator Chip—μPC)	Outdoor.	This wearable has potential applications, including hazardous VOC exposure monitoring in occupational hazard assessment for certain professions, for example in industries	
[102]	15 volatile organic compounds (Microfabricated Preconcentrator Chip—μPC)	Thermal: temperature and humidity data. GPS. (Microfabricated Preconcentrator Chip—μPC)	Outdoor.	This device can be useful to collect personal exposure data for epidemiology studies, and may be especially relevant for asthma studies involving VOC triggers.	Size and weight
[104]	TVOCs	Thermal: temperature and humidity data.	Indoor/Outdoor	The study demonstrate the potential of the device for environmental monitoring, individual health (biomarkers detection) and population health studies.	
[105]	VOCs, CO		Indoor/Outdoor	The proposed VOC device is adequate to characterize personal exposure in many real-world scenarios and is applicable for personal daily use.	
[106]	VOCs, CO		Indoor/Outdoor	This integrated approach offers a cost-effective and reliable platform for personal exposure assessment.	
[107]	Ozone (MiCS-2614), TVOCs (SGPC3)	Thermal: Air Temperature and Relative humidity (SHT30-DIS-B). Accelerometer for activity level (NXP MMA8652FC)	Indoor/Outdoor	This work shows that an inexpensive and compact wearable device can be built, and it also reveals additional improvement, particularly in shortening of response time, reducing cross interference from unintended gases present in air, and determining when recalibration is necessary are all important for a device intended for use under real-world scenarios.	
[45]	CO, NO_2_, O_3_, and SO_2_.	Thermal: Air Temperature and Relative humidity. Visual: UV. Accelerometer to track a user’s activity.	Indoor/Outdoor	Outdoor and indoor air quality assessment.	The system had not been fully developed in terms of the Internet connectivity and smartphone application.
[110]	Particulate matter—PM2.5 (Ultrasonic Personal Aerosol Sampler—UPAS)		Indoor. Rural Honduran women who use wood-burning cookstoves.	Using the UPAS as a personal exposure monitor for household air pollution studies.	
[69]	Ozone, Sulfur Dioxide, Volatile Organic Compounds (Eco-Mini)	Acoustic: Sound pressure level. Thermal: relative humidity, temperature. Visual: ambient light color balance, 3-axis accelerometer and GPS location (Eco-Mini)	Indoor/Outdoor	New forms of low-cost portable monitors have begun to emerge that enable the collection of higher spatial density “crowd sourced” data.	
[112]	PM_1_, PM_2.5_, PM_10_ (Alphasense OPC-N2)	Relative humidity and air temperature (DHT22) because the velocities of the particles change under varying temperature and humidity conditions according to the Ideal Gas Law and it is important for the system to be examined separately under each variable condition in a controlled manner.	Indoor/Outdoor	The results of this study show that a reliable wireless environmental monitoring system for real-time remote air quality and health monitoring applications is feasible.	battery size and lifespan
[121]	NO_2_, CO		Indoor. Laboratory test	A low-power sensing platform for health and environment monitoring with embedded sensors and external sensors was developed and tested.	
[113]	PM_1_, PM_2.5_, PM_10_		Indoor (second and fourth floor flats, cafes, cars, restaurants, underground metro). Outdoor.	The study showed that the residences’ air quality was determined by the type and intensity of outdoor sources and their vertical distance from the street. Indoor activities such as cooking and cleaning further increased PM levels and formulated the air quality, while particulate accumulation was evident.	
[125]	CO_2_ (COZIR-GC0012)	Thermal: air temperature, relative humidity (BME280). Pressure (BME280). Visual: illuminance (TSL2591)	Indoor Environmental Quality assessment	The proposed work provides an effective and reliable solution for long-term monitoring that presents many opportunities in safety related monitoring applications, by using a wireless sensor network for wearable environmental monitoring.	
[122]	NO_2_, CO and SO_2_ additionally (Ubiqsens)	Thermal: air temperature, relative humidity. Pressure. Acoustic: noise level. Accelerometer, Gyroscope, Magnetometer. (Ubiqsense)	Indoor. Laboratory test.	The wearables for gas monitoring under real laboratory conditions show high potential.	
[114]	PM_2.5_	GPS	Indoor/Outdoor. Children in their everyday environments	The overall findings of this study indicate that both the choice of transport mode and the type of neighborhood built environment have an influence on children’s exposure to fine particulate matter during their school commute. This study has revealed how the school commute can be a target for various mitigation strategies by city planners, transportation engineers, school boards, and parents aimed at reducing the burden of air pollution on children. The finding that mean PM_2.5_ exposure was lowest among those who walked to and from school and higher among those who were driven should be of particular interest to public health professionals promoting active school travel.	
[111]	PM_2.5_, PM_10_		Indoor. Two Buddhist temples in Tai-Chung	This study was conducted to assess worshippers’ exposure to PM_10_ and PM_2.5_ in two different types of Buddhist temples in Tai-Chung. As a result, people should spend less time in Buddhist temples, choose a well-ventilated temple, or avoid visiting temples on the 1st and 15th days of the lunar month.	
[115]	PM_2.5_	GPS, Wearable cameras.	Indoor (household). Outdoor.	The study investigates the relationship between particulate air pollution from outdoor and household sources with markers of atherosclerosis in Telangana, India.	
[123]	NO_2_, CO	Thermal: temperature, humidity. Air pressure.	Indoor/Outdoor	In this work, a preliminary innovative working prototype of a wrist-worn watch health sensor monitoring system for environment air quality (physical and chemical parameters) was introduced.	
[68]	CO (DDScientific GD) and CO_2_ (Dynament MSH-P-CO2/NC) concentration [ppm], VOCs [kOhm] (Figaro TGS8100)	Air Temperature [°C], Relative Humidity [%] (BME280). Wind Direction [°], Wind Speed [m/s] (LCJ-CAPTEURS CV07-OEM). Surface Temperature [°C] (Flir vue Pro R). Visual: Illuminance [lx], Global solar radiance [W/m^2^] (SP-215)	Outdoor. Wearable used to study pedestrians’ exposure to urban environmental conditions	A miniaturized microclimate station, specifically tailored to be worn while walking or biking and therefore to collect data according to the pedestrian perspective in anthropized areas.	
[116]	PM_2.5_	GPS, Wearable cameras.	Indoor (household). Outdoor.	The study investigates the relationship between particulate air pollution from outdoor and household sources with markers of atherosclerosis in Telangana, India.	
[76]	ultrafine particles (UFP, <100 nm diameter)	Noise exposure (NEATVIBEwear)	Indoor. Transit, home, school	Personal measurements of UFP (ultrafine particle) and noise will enable researchers to investigate the independent and/or joint-effects of these health-relevant environmental exposures.	NEATVIBEwear, in its current release, does not allow for 1/3-octave band or spectral measurements of traffic noise.
[126]	CO and CO_2_ (WE-Safe)	Thermal: Air Temperature and Relative humidity. Visual: UV. (WE-Safe)	Indoor/Outdoor	This paper presented a wearable for people working in extreme and harsh environments when they are not in safe zones. Such an IoT platform will present new opportunities for saving lives or preventing health issues etc.	
[108]	O_3_, NO_x_, CO_x_, and VOCs	Thermal: Air Temperature and Relative humidity. Visual: UV, ambient light.	Indoor/Outdoor	The aim of this study is to develop a wearable environmental monitoring system (WEMS), an IoT device, to measure environmental variables. Such an IoT platform will present new opportunities for saving lives or preventing health issues, etc.	
[117]	PM (with 6 particle size channels: 0.3 µm, 0.5 µm, 1.0 µm, 2.5 µm, 5.0 µm, 10 µm) (CEM DT-9881)	Thermal: Air Temperature and Relative humidity (CEM DT-9881). Heart rate and the cadence of walking or cycling speed (Wahoo Fitness sensor TICKR RUN). Subjective data about the feelings of air quality and the symptoms (e.g., cough, sore throat, eye itchiness, etc.) reported via the smartphone App CrowdAir	Outdoor	The data collection operation and data analysis results demonstrate the feasibility of the adopted methodology and the developed platform to identify the correlations among air quality indicators, participants’ subjective feelings of air quality, physical activity status measured by wearable sensors, and reported symptoms.	
[79]	PM_2.5_ an PM_10_ (PLANTOWER PM5003).	Thermal: air humidity, temperature (PLANTOWER PM5003). Noise [db] (LENA—Language Environment Analysis System)	Indoor. School, Home.	Preliminary results indicate that it is feasible to gather personal Particulate Matter (PM_2.5_ and PM_10_), language, and noise data, cognitive assessments, and biospecimens from a sample of 3-4-year-old children.	
[88]	Combustible gas concentration (NTC IGD)	Air Temperature [°C] (ATxmega16E5 in-built temperature sensor)	Indoor. Employee state monitoring	A wearable wireless sensor system can be attached to a uniform and used for monitoring combustible gas concentration and temperature. The main feature of the wireless system is the possibility to be activated remotely by an RF control signal.	
[127]	explosive and nerve-agent		Indoor. Laboratory test	The study describes a wireless wearable ring-based sensor system for rapid electrochemical monitoring of explosive and nerve-agent threats in vapor and liquid phases. Overall, such a fully integrated ring-based wearable platform holds considerable promise for meeting rapidly growing defense and security sensing needs.	
[90]	Ozone, particulate matter, CO (EnviroSensor 2.0).	Thermal: Temperature [°C], Relative Humidity [%]. Position: Latitude and longitude (EnviroSensor 2.0).	Laboratory test	Portable air quality sensors have the potential to fill in the gap left by traditional air pollution monitoring.	Power draw of the sensor
[124]	CO_2_ (COZIR-AJ-5000)	Thermal: Temperature [°C], Relative Humidity [%] (SHT21). Air pressure (BMP180). Acceleration and angular velocity (MPU-6050).	Indoor/Outdoor	The device capability has been assessed using a testing strategy that included three scenarios: indoor measurements, outdoor measurements, and indoor-outdoor measurements. It has shown promising results and a good match with the values found in the literature.	
[91]	CO_2_ [ppm]	Temperature [°C], Relative Humidity [%] (SHT11)	Indoor.	This indoor smart environment monitoring system for safety applications is based on custom wearable sensor nodes, connected to a static WSN. The system has been developed for a hazardous gas environment, but could be applied to a number of other safety applications or in other areas such as the tracking of medical devices in a hospital.	
[92]	Ozone.	Thermal: Temperature [°C], Relative Humidity [%]. Biometric parameters: Herat rate, respiratory rate and expiratory airflow, skin impedance. Acceleration.	Indoor.	The system consists of a wristband, a chest patch, and a handheld spirometer.	
[93]	distinguishing and counting differently sized particles (My Part)	Thermal: Temperature [°C], Relative Humidity [%] (My Part).	Indoor/Outdoor.	The study reports the results of a preliminary user study conducted to evaluate the experience of using the overall system in terms of accuracy, low cost, and portability.	
[95]	NO_2_, O_3_ and CO	Thermal: Temperature [°C], Relative Humidity [%].	Outdoor.	The author reports the design and initial deployment of the Citisense mobile air quality sensing system.	
[103]	VOCs (Figaro TG2620 sensor)		Outdoor.	The authors presented WearAir, an expressive T-shirt to sense the surrounding air quality, as indicated by the measured volatile organic compounds. It can motivate others to study ways to convey environmental information more effectively.	
[118]	PM_2.5_ (RTI MicroPEM v3.2b)	Thermal: Temperature [°C], Relative Humidity [%]. Acceleration. (RTI MicroPEM v3.2b).	Outdoor.	Authors developed a machine learning model for identifying periods of bicycling activity using passively collected data from the MicroPEM wearable. This finding helps strengthen the case for using wearable monitors in exposure assessment studies, as it becomes possible to estimate potential inhaled doses if personal exposure data is combined with respiration rate.	
[84]	Polycyclic aromatic hydrocarbons—PAHs, oxygenated PAHs—OPAHs, Polychlorinated biphenyls—PCBs, Pesticides, organophophorous flame retardants—OPFRs. (MyExposome). Surface ozone content—SOC (2BTech Personal Ozone Monitor)	Thermal: Air Temperature and relative humidity (Model HTR-170). Visual: UV (UV dosimeter badge).	Outdoor.	Polycyclic aromatic hydrocarbons (PAH) indicate possible causes of hair damages. This is the first meteorotropic study of its kind, combining environmental aggressors related to hair damage, opening new research hypothesis further studies on exposome.	
[72]	CO and NO_2_	Thermal: air humidity, temperature. Acoustic: Sound pressure level [db] (MLMS-EMGN-4.0). Air pressure	Indoor. Laboratory	This device measures several physical and chemical environmental parameters that are known to be hazardous.	
[75]	NO_2_, CO	Acoustic: Sound level [db]. Thermal: air temperature and relative humidity. UV. Biometrics: skin temperature, heart rate. Motion activity (9 Degree of Freedom(DoF)) motion tracking by accelerometer, gyroscope and magnetometer.	Indoor/Outdoor	Wrist-worn devices can integrate parameters from the environmental, behavioral, and physiological domains.	
[78]	Styrene exposure derived by biological monitoring in saliva and urine	Noise levels (Quest DLX-1)	Indoor. Workers employed in molding and in artifacts refining	A significant negative correlation was found between otoacoustic emission levels and the concentration of the styrene exposure.	
